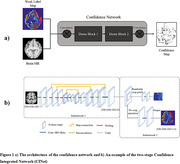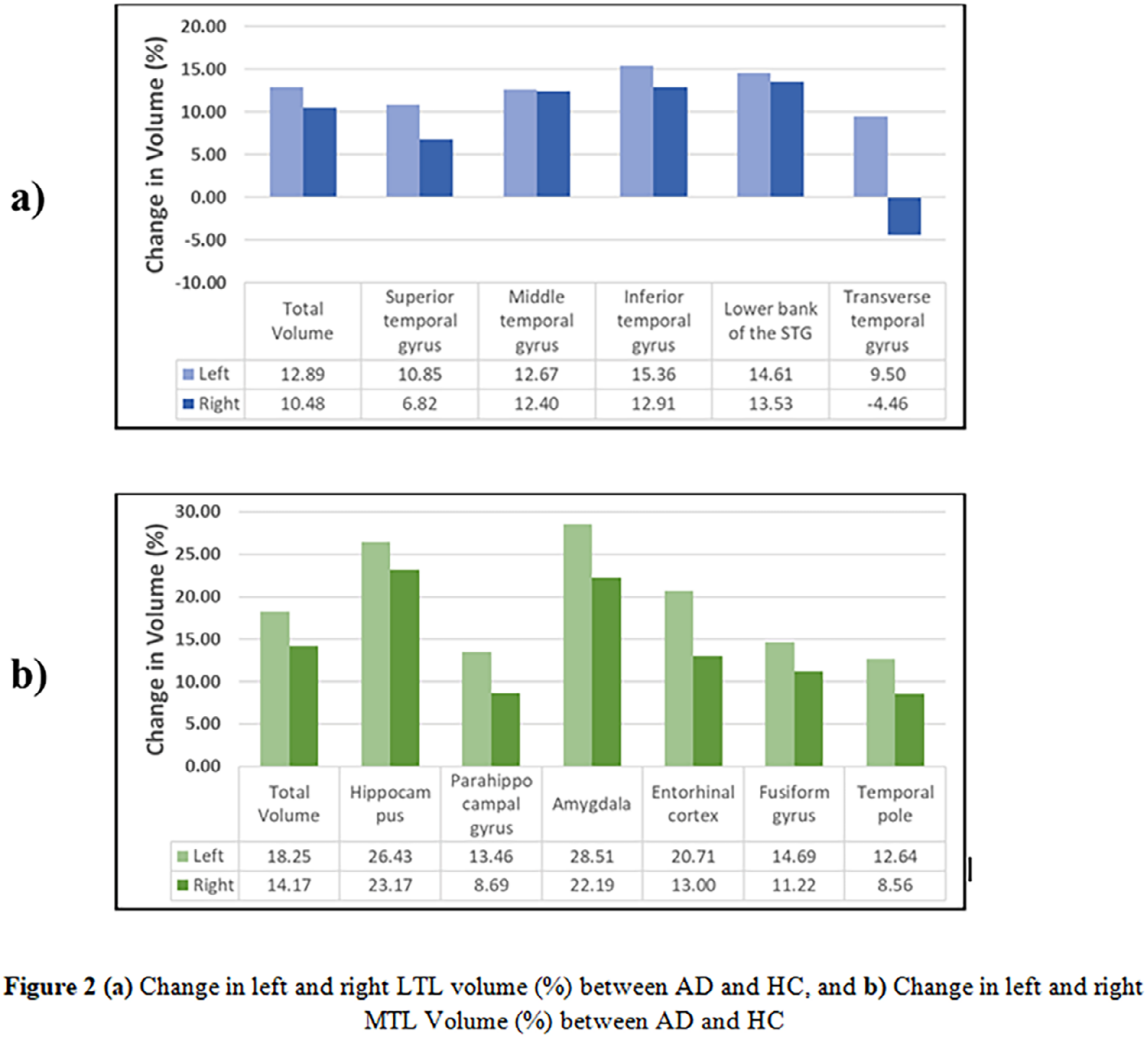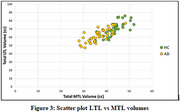# Automated quantification of lateral and medial temporal lobe volumes for improved diagnosis of early Alzheimer's disease

**DOI:** 10.1002/alz70856_102485

**Published:** 2025-12-26

**Authors:** Marufjon Salokhiddinov, Dileep Kumar, Dharmesh Singh, Munojat Ismailova, Akash Gandhamal

**Affiliations:** ^1^ Tashkent Medical Academy, Tashkent, Tashkent, Uzbekistan; ^2^ Central Research Institute, Shanghai, Shanghai, China; ^3^ Cenral Research Institute, Delhi, Delhi, India

## Abstract

**Background:**

The purpose of this study is to evaluate the importance of automated lateral and medial temporal volume measurement techniques for the early diagnosis of Alzheimer's disease.

**Method:**

This study employed 3T MPRAGE MRI scans from participants enrolled in the ADNI – Phase 3 : 39 HC (healthy control) and 39 individuals diagnosed with mild AD. Cascaded weakly supervised confidence integration network (CINet) developed by United Imaging Intelligence Shanghai, China for automating volumetric quantification of distinct brain regions through brain MR image parcellation. This study focuses on analysing atrophies in the MTL and LTL regions in patients with mild AD. The examination includes not only the entire MTL (Medial Temporal Lobe) and LTL (Lateral Temporal Lobe) regions but also their respective sub‐divisions.

**Result:**

For all subjects, the total volume of the left LTL in AD patients showed a significant (*p* <0.05) reduction of ∼13% compared to HC. According the gender‐based analysis, the average volume in the left LTL was 37.85 ± 5.28 cc for HC males and 32.90 ± 4.55 cc for males with AD, and for HC female was 33.65 ± 4.71 cc and 29.25 ± 3.85 cc for females with AD. The volume loss of the left LTL in subjects with AD was ∼ 13% for both males and females. The total volume of the right LTL in AD patients showed a significant (*p* <0.05) reduction of ∼10.50% compared to HC for all subjects. The average volume in the right LTL was 36.10 ± 4.37 cc for HC males and 32.30 ± 3.57 cc for males with AD, and for HC female was 32.15 ± 3.29 cc and 28.80 ± 3.79 cc for females with AD. Both males and females with AD showed ∼10.50% volume reductions in the right LTL.

**Conclusion:**

Higher volume loss is observed in the left MTL, and LTL regions compared to the right, indicating an asymmetric impact in mild AD. The study underscores the significance of automated techniques for AD diagnosis and monitoring disease progression, contributing valuable insights for potential early interventions.